# Preventing distal stent deflection using a sheath-assisted technique for malignant hilar biliary obstruction

**DOI:** 10.1055/a-2779-7471

**Published:** 2026-02-03

**Authors:** Kenichi Haneda, Akihisa Kato, Yusaku Tomita, Yusuke Kito, Tadashi Toyohara, Yasuki Hori, Michihiro Yoshida

**Affiliations:** 1 Department of Gastroenterology and Metabolism, Nagoya City University Graduate School of Medical Sciences, Nagoya, Japan

Endoscopic biliary stent placement for malignant hilar obstruction is a standard but often technically demanding procedure. Severe strictures can make the advancement of a stent difficult, particularly when multiple stents are required. Common strategies include exchanging for a stiffer guidewire or dilating the stricture with a dilator or balloon catheter 1 2 ; however, these may not always be successful. In particular, when inserting a plastic stent, stent deflection in the distal bile duct can dissipate the pushing force, preventing the stent from crossing the stricture.

We report a case in which an endoscopic sheath was used to prevent distal stent deflection and facilitate the successful plastic stent placement across a tight hilar stricture.

**Fig. 1:**
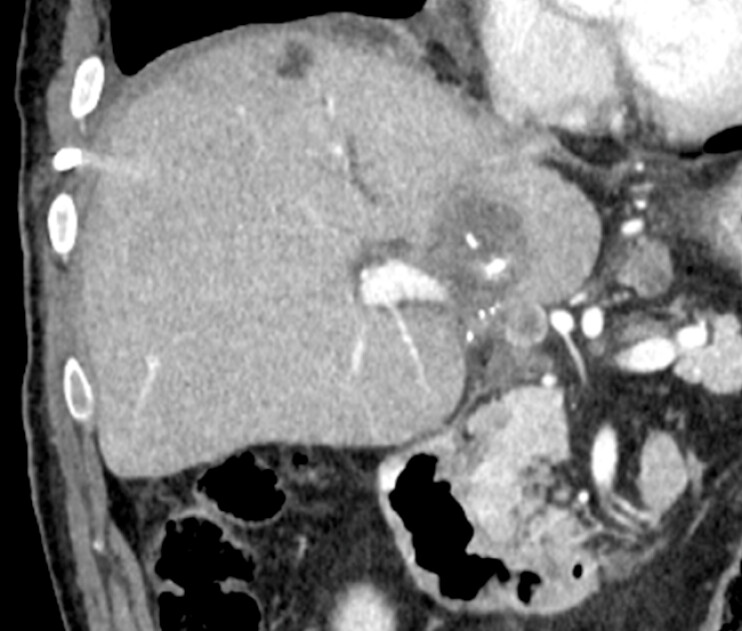
Contrast-enhanced CT revealed recurrent hepatocellular carcinoma in the liver S1. CT, computed tomography.

**Fig. 2:**
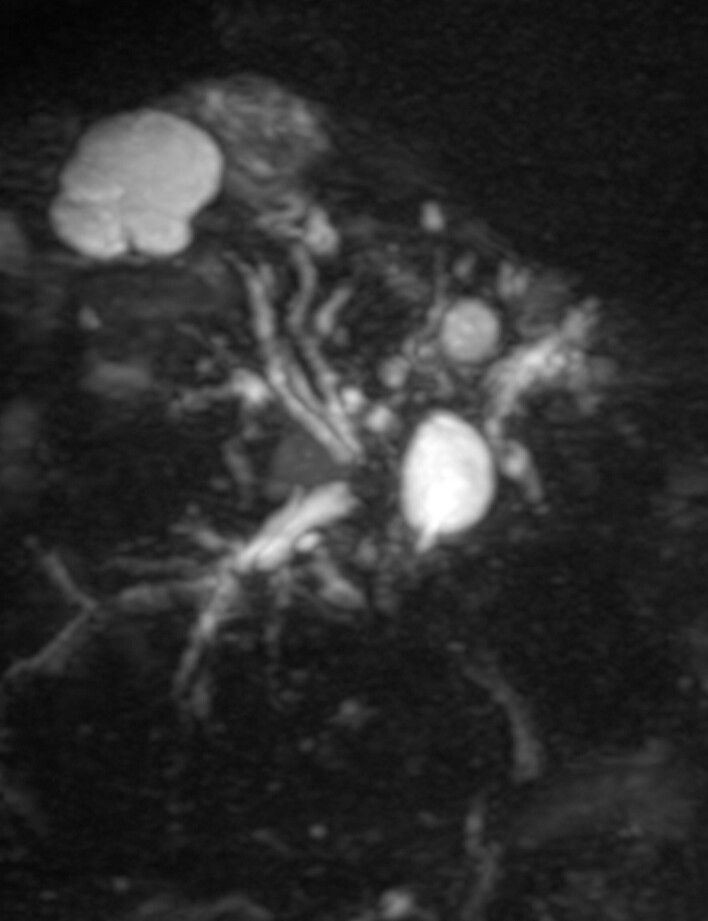
MRCP showed that the anterior and posterior segments of the bile duct were dilated and separated due to HCC in S1. HCC, hepatocellular carcinoma; MRCP, magnetic resonance cholangiopancreatography.

A 70-year-old man with a history of left hepatic lobectomy for hepatocellular carcinoma developed recurrent diseases in segment 1 of the liver ( Fig. 1 ). Magnetic resonance cholangiopancreatography demonstrated hilar bile duct obstruction due to the tumor in S1, resulting in dilatation of the anterior and posterior segmental ducts ( Fig. 2 ).

Endoscopic biliary stenting was performed. Guidewires were successfully placed in both segmental ducts, and a 7-Fr plastic inside stent was inserted into the posterior duct. However, attempts to place another 7-Fr plastic stent into the anterior duct failed because of distal stent deflection, even after exchanging the guidewire from 0.025-inch to 0.035-inch.

**Fig. 3:**
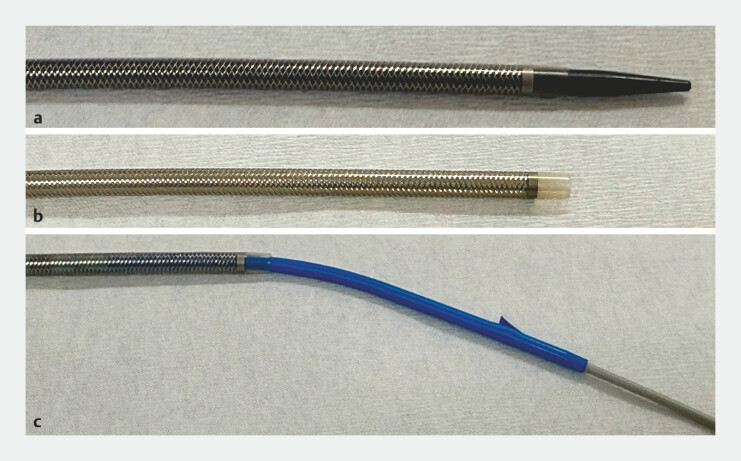
**a** An appearance of the modified EndoSheather with an enlarged sheath diameter (an inner diameter of 8.4 Fr and an outer diameter of 10 Fr). **b** Outer sheath of EndoSheather. **c** The 7-Fr integrated plastic stent in the outer sheath of EndoSheather.

**Fig. 4:**
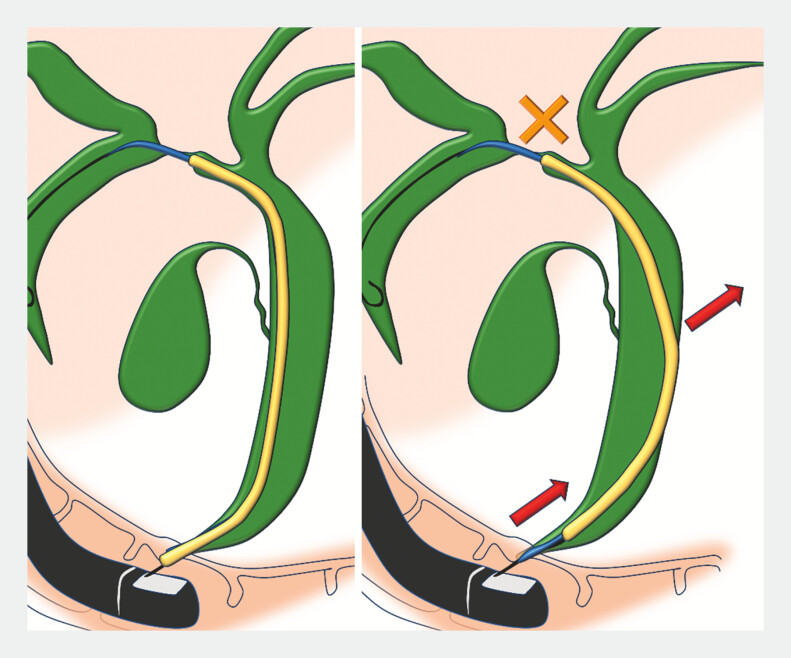
The 7-Fr plastic stent deflected in the distal bile duct, preventing it from transmitting enough forces to penetrate the stricture.

**Fig. 5:**
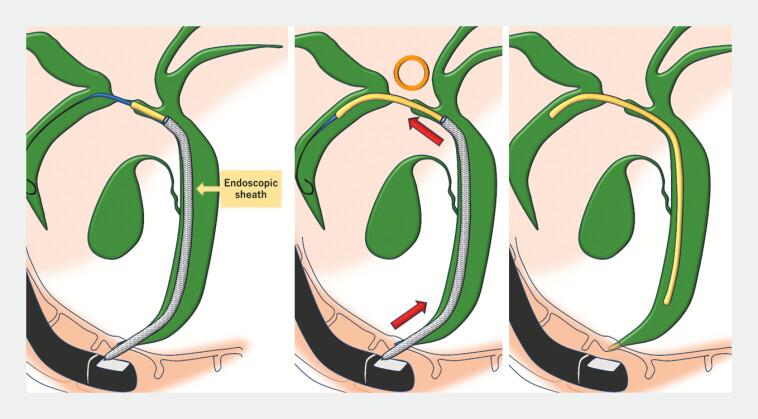
EndoSheather was advanced over the guidewire to the hilar bile duct. The sheath prevented the plastic stent from bending in the distal bile duct, allowing the force required to insert the 7-Fr stent to be transmitted sufficiently to the stricture, enabling the stent to be placed beyond the stricture.

To overcome this, a large-caliber version of EndoSheather (Piolax, Inc., Yokohama, Japan, Fig. 3 ) 3 4 5 was advanced over the guidewire to the hilar bile duct. After removing the inner catheter, the 7-Fr plastic stent was inserted through the outer sheath. This approach prevented distal stent deflection and enabled the stent to cross the stricture smoothly for successful deployment ( Fig. 4 , Fig. 5 , Video 1 ).

The endoscopic sheath assisted in preventing the distal deflection of the plastic stent and enabled its smooth insertion across the tight hilar stricture.Video 1

When plastic stent deflection in the distal bile duct impedes insertion during hilar stenting, the use of an endoscopic sheath can provide effective axial support, reduce deflection, and facilitate smooth stent placement.

Endoscopy_UCTN_Code_TTT_1AR_2AZ
